# Clinical and cognitive correlates of employment among patients with schizophrenia: a cross-sectional study in Malaysia

**DOI:** 10.1186/1752-4458-5-14

**Published:** 2011-05-30

**Authors:** Marhani Midin, Rosdinom Razali, Ruzanna ZamZam, Aaron Fernandez, Lim C Hum, Shamsul A Shah, Rozhan SM Radzi, Hazli Zakaria, Aishvarya Sinniah

**Affiliations:** 1 Department of Psychiatry, Faculty of Medicine, Universiti Kebangsaan Malaysia, Kuala Lumpur 56000, Malaysia; 2 Department of Psychiatry, Kuala Lumpur Hospital, Kuala Lumpur 50586, Malaysia; 3 Department of Psychiatry, Ampang Hospital, Kuala Lumpur 68000, Malaysia; 4 Department of Community Health, Faculty of Medicine, Kuala Lumpur 56000, Malaysia; 5 ExxonMobil Exploration and Production Malaysia Inc

**Keywords:** schizophrenia, employment, cognitive function, Malaysia

## Abstract

**Background:**

Gainful employment is one major area of functioning which is becoming an important goal in psychiatric rehabilitation of patients with schizophrenia. Studies in western countries are pointing to evidence that certain sociodemographic and clinical factors may contribute to employment outcomes in this group of people. However, the area is still largely unexplored in Malaysia. The aim of this study was to examine the sociodemographic, clinical and cognitive correlates of employment status among patients with Schizophrenia.

**Methods:**

This was a cross-sectional study. All participants who fulfilled the requirements of the study according to the inclusion and exclusion criteria were enrolled. Study instruments included a demographic data questionnaire, Positive and Negative Symptom Scale (PANSS), Trail Making Tests, Rey's Auditory Verbal Learning Test (RAVLT) and Digit Span. Bivariate analyses were done using chi-square for categorical data and t-test for continuous data and multiple logistic regression analysis was done to identify predictors of employment status.

**Results:**

A total of 95 participants who fulfilled the inclusion criteria were enrolled into the study. Among the sociodemographic, clinical and cognitive variables studied marital status, educational level, mean scores of negative symptoms, Digit Span and RAVLT and Trail Making Tests were found to show significant association with employment status on bivariate analyses. However, when entered into a logistic regression model, only cognitive variables ie. Trail A and B, Digit Span and RAVLT were significant predictors of employment status.

**Conclusions:**

The results from this study support the role of cognitive function, particularly, attention, working memory and executive functioning on attaining and maintaining employment in persons with schizophrenia as measured by the RAVLT, Digit Span and Trail Making Tests. These findings may act as preliminary evidence suggesting the importance of integrating cognitive rehabilitation in the psychosocial rehabilitation program for patients with schizophrenia in Malaysia.

## Introduction

Long-term goals in treatment of patients with schizophrenia had long been extended beyond symptom remission to recovery of functioning. Employment as one of the major areas of functioning has attracted a lot of interest among researchers in recent years as a result of the huge economic cost of unemployment and the development of certain employment models for this group of patients. Gainful employment has increasingly been taken as one of the goals in psychiatric rehabilitation.

Employment has repeatedly been shown to have an important role in patients' recovery from any severe mental illness (SMI), particularly schizophrenia [1-9]. Employment not only provides income, it also improves activity and social contacts in patients with schizophrenia and improves self esteem, quality of life, and perhaps leads to better treatment compliance, symptom reduction and insight [1,2,8-10]. Work or employment is seen both as a treatment outcome and as a highly effective treatment modality in enhancing meaningful community integration [11] as it provides a way of social inclusion and participation as enjoyed by non-mentally ill persons [12]. Employment in patients with schizophrenia indeed needs further exploration.

There have been many studies looking into the role of symptoms and cognitive impairment in employment outcomes in western countries [7,13-25]. Most studies seem to be pointing to evidence that cognitive functions have significant association with employment outcomes [7,25-29]. While the relationship between cognitive function and employment is quite established, results on the role of symptoms in employment have been inconsistent [29] with more studies showing negative symptoms but not positive symptoms as significant correlates of employment [7,26].

In Malaysia, the whole area of employment of patients with schizophrenia is still unexplored. It is important that this aspect of schizophrenia be examined in the local context so that further development in the emerging employment programs in the country would be guided by evidence.

### Objectives of study

The aim of this study was to examine the sociodemographic, symptom and cognitive correlates of employment among patients with Schizophrenia. The symptom and cognitive correlates included: 1. Symptom type and severity as measured by PANSS; 2. Cognitive impairment as measured by RAVLT, Trail Making A & B and Digit Span.

## Methodology

This comparative cross-sectional study was done at the Psychiatric and Mental Health Clinic of Kuala Lumpur Hospital (KLH). The hospital has the largest number of patients with schizophrenia receiving care on an outpatient basis in Malaysia. The clinic has a patient load of approximately 100 patients per day with most patients having a diagnosis of schizophrenia.

The subjects were patients who fulfilled the DSM IV-TR criteria for the Schizophrenia, diagnosed by the attending psychiatrists and were confirmed by a research psychiatrist, AF, who collected the data. Other inclusion criteria included: 1) being in stable phase of schizophrenia as defined as having no hospitalizations, changes in housing or changes in medication dosages in the past 4 months; 2) age between 18 and 60 years. Exclusion criteria included: 1) having co-morbid substance abuse; 2) having central nervous system diseases (e.g. epilepsy, head injury, cerebro-vascular disease or dementia); 3) refused consent. Purposive sampling method was employed and all participants who fulfilled the requirements of the study according to the inclusion and exclusion criteria were enrolled after informed consent was given. Respondents filled in the Demographic Data Questionnaire before they were assessed using the PANSS. AF completed a training module on the PANNS and the neurocognitive tests.

A total of 150 subjects were screened for eligibility over a period of one month, of which, 55 fulfilled the exclusion criteria, leaving 95 patients who fulfilled the inclusion criteria being enrolled into this study. Exclusion from the study was due to the following reasons: one was too ill psychiatrically to complete the interview; seven had ongoing substance abuse; 14 fulfilled diagnosis of bipolar mood disorder; 12 had a diagnosis of psychotic depression; seven had a diagnosis of drug-induced psychosis; eight had epilepsy or mental retardation and six refused consent. There was no significant difference between the age and gender distributions of the respondents and non-respondents.

### Study instruments

#### a) Demographic data questionnaire

The demographic questionnaire gathered information on gender, age, ethnicity, marital status, education level, duration of illness, employment status and occupational class. Employment in this study was defined according to the provisions of the law which means "any service performed for remuneration under a contract of hire which creates the employer and employee relationship". This included: a) full-time employment-workers scheduled to work between 35 and 40 hours per week; b) part-time employment-permanent workers with less than full time working hours; c) temporary employment for only a short period of time, such as for some special project. In this study, the type of employment was further categorized into the same occupational classes as used in the statistics of Malaysian population: professional and administration; clerical; sales; service; and manual labour.

#### b) PANSS

This widely used 30-item instrument measures three dimension of schizophrenia, namely positive, negative and general psychopathology [30]. It has been repeatedly shown to have criterion-related validity with antecedent, genealogical, and concurrent measures, predictive validity, drug sensitivity, and utility for both typological and dimensional assessment [30].

The positive scale consists of 7 items measuring symptoms such as hallucinations and delusions; the negative scale includes 7 items assessing symptoms such as blunted affect and apathetic social withdrawal; and a general psychopathology scale includes 16 items assessing severity of schizophrenic illness. Symptom severity is assessed on a 7-point scale ranging from 1 (*absent*) to 7 (*extreme*). The scale has good internal reliability for the positive, negative, and general psychopathology scales [30], inter-rater reliability and test-retest reliability [31].

#### c) Trail Making Test

The Trail Making Test consists of two measures: Trails A and Trails B. Trails A is a test of psychomotor speed that requires subjects to connect numbers in ascending order. Trail Making Test B is a measure of combined visual search, psychomotor speed, and cognitive flexibility. Part B of the Trail Making Test assesses the shifting and maintenance of response set by requiring individuals to sequentially alternate between alpha-numeric sequences (e.g., 1-A-2-B-3-C). Most reports of reliability have been between .60 and .90 s The Trail Making Test is sensitive to CNS deficits, whether it is early stages of attention, head injury as well as neuropsychological deficits such as have been reported in schizophrenia [32]. Age-standardized scores were used to demarcate between normal and abnormal scores.

#### d) Rey's Auditory Verbal Learning Test (RAVLT)

In this research, RAVLT was used to assess short-term auditory verbal memory. The standard administration format of the RAVLT consists of reading a list of 15 unrelated words (List A) repeated over five different trials. In this study, the score following the fifth trial which indicates best learning was used for analysis.

#### e) Digit Span

Digit span measures several psychological parameters as follows: immediate recall, both forwards and backwards; ability to shift thought patterns; attention and concentration; auditory short term memory; and auditory sequencing. Subjects were given sets of digits to repeat initially forwards then backwards. In doing so, they were required to accurately encode the information as well as accurately recall, sequence and vocalize the auditory information. The combined raw scores of digit forward and backward were calculated and this was converted to a scaled score and used for analysis.

### Statistical analysis

Data were analysed using the Statistical package for Social Sciences (SPSS) (version 15) computer program. Employment was the dependent variable and was treated as a binary variable (employed versus unemployed). Bivariate analyses were done using chi-square for categorical data and t-test for continuous data. Mean and standard deviation (SD) were used to describe continuous variables.

Multiple logistic regression analysis was done to predict significant independent variables for employment status.

### Ethical consideration

This research project was approved by the Research Ethics Committee of the Faculty of Medicine UKM and the Medical Director of HKL. The purpose of the study was explained and consent was obtained from the subjects. All the respondents were assured of the confidentiality of the data collected. All the subjects were included on voluntary basis.

## Results

Sociodemographic, employment, clinical and cognitive characteristics of the subjects are shown in Table 1. From bivariate analyses, there were significant differences found in several variables between the employed and unemployed groups (Table 2 and 3). About 70% of the married subjects were in the employed group (p = 0.02, OR of 3.10 [95%CI 1.19-8.11]). About 80% of participants with education of higher than secondary school level were in the employed group (p = 0.02, OR 4.36 [95%CI 1.13-16.80]). On the PANSS scores, only mean for PANSS scores for negative symptoms was found to be significantly different in the 2 groups (p = 0.005). Lower Negative PANNS scores were associated with being employed (Table 3). There were significant differences in scores of all the cognitive tests between the 2 groups (Table 3), all with p values < 0.001. Normal scores in Trail A and B and higher Digit Span and RAVLT scores were associated with being employed.

**Table 1:** Characteristics of the Respondents (N = 95)

Patient Characteristics		Mean (SD)	N (%)
**Age (years)**		37.7(8.6)	
**Gender**	Male		65 (68.4%)
	Female		30 (31.6%)
**Ethnicity**			
	Malay		56 (58.9%)
	Chinese		32 (33.7%)
	Indian		6 (6.3%)
	Others		1 (1.1%)
**Marital Status**			
	Single		64 (67.4%)
	Married		26 (27.4%)
	Divorced		4 (4.2%)
	Separated		1 (1.1%)
	Widowed		0 (0%)
**Educational Level**			
	No Formal Schooling		0 (0%)
	Primary School		18 (18.9%)
	Secondary School		63 (66.3%)
	STPM/Diploma		11 (11.6%)
	Degree		3 (3.2%)
**Employment**	Employed		48 (50.5%)
	Not Employed		47 (49.5%)
**Employment Class**	Professional and administration		6 (12.5%)
	Clerical		3 (6.2%)
	Sales		7 (14.6%)
	Service		13(27.1%)
	Manual labour		19(39.6%)
**Duration of illness (years)**		11.09 (8.2)	
**PANSS scores**	Total	79.17(14.41)	
	Positive symptoms	14.09(4.50)	
	Negative symptoms	23.56(4.98)	
	General psychopathology	34.02(4.05)	
			
**Cognitive tests scores**	Trail A	48.11(23.25)	
	Trail B	122.02(76.82)	
	Digit span	7.52(2.42)	
	RAVLT	8.48(2.76)	

N = number of subjects, % = percentage, SD = standard deviation

**Table 2:** Demographic variables and employment status from bivariate analyses (N = 95)

Variables	Employed	Unemployed	P value	OR(95% CI)
	N(%)	N(%)		
**Age (years)**				
Mean(SD)	38.83(9.14)	36.58(7.85)	0.20	-
**Gender**				
Male (65)	33(50.8)	32(49.2)	0.56	0.97(0.41-2.30)
Female (30)	15(50.0)	15(50.0)		
**Ethnicity**				
Malay (56)	25(44.6)	31(55.4)	0.12	1.78(7827-4.07)
Non-Malay (39)	23(59.0)	16(41.0)		
**Marital Status**				
Married (26)	18(69.2)	8(30.8)	0.02 *	3.10(1.19-8.11)
Single/divorced/separated/widowed (69)	29(42.0)	40(58.0)		
**Educational Level **^b^				
≤ Secondary School (81)	37(46.7)	44(54.3)	0.02 *	4.36(1.13-
> Secondary School (14)	11(78.6)	3(21.4)		16.80)

SD = standard deviation, N = number of subjects, % = percentage, CI = confidence interval, OR = odds ratio.

**Table 3:** Clinical and cognitive variables and employment status from bivariate analyses (N = 95)

Variables	Employed	Unemployed	p value
	Mean (SD)/N(%)	Mean (SD)/N(%)	
**Duration of illness (years)**	12.40(8.73)	9.80(7.51)	0.13
			
**PANSS scores**			
Total	78.06(15.16)	80.30(13.67)	0.45
Positive symptoms	14.16(4.70)	14.02(4.33)	0.88
Negative symptoms	22.60(5.48)	25.53(4.49)	0.005*
			
**Cognitive tests scores**			
Trail A			
Normal	36(72)	14(28)	
Not normal	12(26.7)	33(73.3)	< 0.001*
Trail B			
Normal	39(73.6)	14(26.4)	< 0.001*
Not normal	9(21.4)	33(78.6)	
Digit span	8.56(2.41)	6.46(1.94)	< 0.001*
RAVLT	9.38(2.72)	7.57(2.52)	0.001*

SD = standard deviation, CI = confidence interval

When these variables were entered into a stepwise logistic regression model to predict employment status; marital status, education level and PANSS negative symptoms were removed from the model leaving only Trail A and B, Digit Span and RAVLT scores as significant predictors of employment status among patients with schizophrenia (Table 4).

**Table 4:** Predictors of unemployment status from binary logistic regression analysis

Variables	B	OR (95%CI)	p value
**Marital status**	-0.92	0.40(0.11-1.38)	0.150
**Education**	1.24	3.45(0.84-14.21)	0.090
**PANSS Negative symptoms**	0.03	1.03(0.92-1.15)	0.610
**Trail A**	1.37	3.97(1.24-12.73)	0.020*
**Trail B**	1.28	3.58(1.15-11.13)	0.027*
**Digit span**	0.32	1.34(1.04-1.72)	0.025*
**RAVLT**	0.55	1.05(0.84-1.32)	0.034*

B = beta value, OR = odds ratio, CI = confidence interval

## Discussion

Literature regarding employment in patients with schizophrenia, particularly on factors associated with employment status, is lacking in Malaysia. In this study, among the demographic, clinical and cognitive variables studied; marital status, educational level, mean scores of negative symptoms, Digit Span and RAVLT and Trail Making Tests were found to show significant association with employment status on bivariate analyses. However, when entered into a logistic regression model, only cognitive variables ie. Trail A and B, Digit Span and RAVLT were significant predictors of employment status.

The results from this study support the role of cognitive function, particularly attention, working memory and executive functioning on attaining and maintaining employment in persons with schizophrenia as measured by the RAVLT, Digit Span and Trail Making Tests. These findings are consistent with those from other studies which showed association of cognitive impairment with unemployment [18,28,29,33]. McGurk and Meltzer (2000) [18] also found that those who were employed full time did significantly better on tests of working memory, vigilance and executive functioning than those who were unemployed. This relationship persisted even after education was controlled for. Among the different domains of cognitive function, several specific domains have been shown to be associated with functional outcome more often than the others. A review by Green and colleagues involving 37 studies in this area [28] confirmed an earlier review [27] and concluded that cognitive domains that were most often found to be associated with social function included secondary verbal memory, immediate memory, executive functioning and vigilance.

Besides employment, cognitive impairment is also associated with the broader functional outcomes among patients with schizophrenia [27-29,34]. In a review paper evaluating neurocognitive functions as predictors and correlates of functioning among persons with schizophrenia by Green [27] found strong and consistent associations between cognitive functions and functioning in schizophrenia. Verbal memory, executive functioning and vigilance were found to be associated with different aspects of functional outcome. These findings were again confirmed by Green in a meta-analysis involving a larger number of such studies [28] as well as by other researchers [26,29,35]. With regard to whether different functioning areas are determined by intact specific cognition functions, independent living skills were found to be associated with executive function [36], vocational functioning with attention and verbal memory [37], and employment status was strongly predicted by intact verbal learning and memory, executive functioning as well as overall intelligence [16]. In addition to personal and vocational functioning, social skills were also found to be dependent on certain intact cognitive domains. For example, social problem-solving was found to be associated with executive functioning and attention [38].

In this study, there was no significant association between overall psychopathology, positive or negative symptoms and employment. This result is not inconsistent with similar studies where a strong correlation between psychopathology and employment has not been consistently established. Green et al [28] reported that in most studies, schizophrenic symptoms were not significantly associated with functional outcome. This is particularly more so for positive symptoms as compared to negative symptoms where some studies found negative symptoms but not positive symptoms to be associated with unemployment in schizophrenia [16,20,39-41]. Negative symptoms in schizophrenia are seen by some as the result of cognitive deficits that impair motivation and insight and hinder therapeutic alliance and compliance [42]. In this study, negative symptoms which were found to be associated with employment status through bivariate analysis were removed from the regression model. This may have been partly due to the problem in eleciting accurate information on negative symptoms as at least half of the participants came to the clinic without other informants. There was a possibility of minimization of negative symptoms among some participants as it is understood that there may be social values attached to negative symptoms.

As this was a cross-sectional study, we can only conclude that there is an association between cognition and employment. We are unable to ascertain causation or directionality. It is possible that unemployment is caused by poor cognitive functions or vice versa. Unemployment has a multitude of possible effects on mental health ranging from reduced self-confidence and social isolation to anxiety and depression. These factors may have an adverse influence on cognitive processes. Furthermore, employment calls upon the exercise of cognitive functions by repetition or training, another form of cognitive remediation. It is however more likely that unemployment in patients with schizophrenia is the result of poor cognitive functioning rather than the cause of it.

Other limitations of this study include the reliance of diagnosis of illness on that made from clinical judgement without use of a diagnostic tool which may have affected the accuracy of diagnosis of the participants enrolled in the study. Secondly, this study was done at a tertiary psychiatric setting in the most populous city in Malaysia, therefore, may not represent other patient populations from other parts of the country.

## Conclusions

In conclusion, this study confirmed significant associations between cognitive functions, particularly information processing speed, executive functioning, attention and short-term verbal memory, and employment status among patients suffering from schizophrenia. These findings provide preliminary evidence of the importance of cognitive rehabilitation in psychosocial rehabilitation programs for Malaysian patients with schizophrenia. From the findings we argue that cognitive rehabilitation programs that has commenced in Malaysia [43] should be scaled up as an important part of rehabilitation in treating schizophrenia. In addition, this should be linked to supported employment programs which are also becoming an important component in rehabilitation for these patients in the country.

## Competing interests

The authors declare that they have no competing interests.

## Authors' contributions

AF, RR and LCH were involved in the conceptualization of the study and literature review. AF recruited the subjects and did the data collection. DS was involved in selecting the cognitive tests and training AF in using the tests. SAS and RSMR were involved in the statistical analysis and interpretation of results. MM and RR were involved in review of the manuscript including extensive revision of interpretation and discussion of the results. All authors read and approved the final manuscript.
